# Evaluating the association of common *APOA2 *variants with type 2 diabetes

**DOI:** 10.1186/1471-2350-10-13

**Published:** 2009-02-13

**Authors:** Konsta Duesing, Guillaume Charpentier, Michel Marre, Jean Tichet, Serge Hercberg, Beverley Balkau, Philippe Froguel, Fernando Gibson

**Affiliations:** 1 Genomic Medicine, Imperial College London, Hammersmith Campus, Du Cane Rd, London, W12 0NN, UK; 2 Endocrinology-Diabetology Unit, Corbeil Hospital, Corbeil, France; 3 Endocrinology-Diabetology, Bichat Hospital, Paris, France; 4 INSERM U695, Paris, France; 5 Institut Régional Pour la Santé, Tours, France; 6 Scientific and Technical Institute of Nutrition and Food (ISTNA-CNAM), Institut National de la Santé et de la Recherche Médicale (INSERM) U557, INRA U1125, Paris, France; 7 INSERM U780-IFR69, Villejuif, France; 8 CNRS 8090, Institut de Biologie de Lille, Institut Pasteur, CHU Lille, France

## Abstract

**Background:**

*APOA2 *is a positional and biological candidate gene for type 2 diabetes at the chromosome 1q21-q24 susceptibility locus. The aim of this study was to examine if HapMap phase II tag SNPs in *APOA2 *are associated with type 2 diabetes and quantitative traits in French Caucasian subjects.

**Methods:**

We genotyped the three HapMap phase II tagging SNPs (rs6413453, rs5085 and rs5082) required to capture the common variation spanning the *APOA2 *locus in our type 2 diabetes case-control cohort comprising 3,093 French Caucasian subjects. The association between these variants and quantitative traits was also examined in the normoglycaemic adults of the control cohort. In addition, meta-analysis of publicly available whole genome association data was performed.

**Results:**

None of the *APOA2 *tag SNPs were associated with type 2 diabetes in the French Caucasian case-control cohort (rs6413453, *P *= 0.619; rs5085, *P *= 0.245; rs5082, *P *= 0.591). However, rs5082 was marginally associated with total cholesterol levels (*P *= 0.026) and waist-to-hip ratio (*P *= 0.029). The meta-analysis of data from 12,387 subjects confirmed our finding that common variation at the *APOA2 *locus is not associated with type 2 diabetes.

**Conclusion:**

The available data does not support a role for common variants in *APOA2 *on type 2 diabetes susceptibility or related quantitative traits in Northern Europeans.

## Background

The *APOA2 *gene is located at chromosome 1q23.3, within the 1-LOD support interval of several genome-wide linkage scans for type 2 diabetes [1-3]. The peak of linkage in the French and Utah Caucasian genome-wide linkage scans was defined by the *APOA2 *intragenic microsatellite D1SAPO2 [1,3]. *APOA2 *encodes the apolipoprotein (apo) A-II, the second most abundant protein of the high-density lipoprotein (HDL) particles [4,5].

Transgenic mice overexpressing human *APOA2 *on a standard chow diet displayed lipid profiles similar to that seen in human type 2 diabetes [6,7]. Moreover, plasma human apoA-II levels were positively correlated with blood glucose levels and these animals displayed impaired glucose tolerance [7]. In the human HepG2 cell line and in rat primary hepatocytes, transcription of the human *APOA2 *transgene was upregulated by glucose in a *HNF-4á*-dependent fashion, whereas in streptozotocin-induced diabetic rats *Apoa2 *mRNA levels were not affected [7].

Overexpression of mouse *Apoa2 *contrastingly resulted in elevated levels of HDL and fasting blood glucose levels that were not significantly different from normal [8-10]. However, these mice appeared to be in a state of insulin resistance exhibiting two-fold raised plasma insulin levels, decreased triglyceride hydrolysis and increased fat deposition in adipose tissue, as well as delayed glucose clearance due to slower uptake in skeletal muscle [8]. Taken together, these findings point to a primary lipoprotein disturbance causing the development of several features of insulin resistance.

On the other hand, homozygous *Apoa2 *null-mice had HDL level reductions of 70%, as well as lower free fatty acid, glucose and insulin levels, suggesting they may be insulin hypersensitive [11].

### Previous genetic analysis of *APOA2 *in type 2 diabetes

Elbein and colleagues tested four *APOA2 *variants for association with T2D by TDT in 698 family members and in a very small case-control cohort of 247 subjects [12]. None of the SNPs exhibited association with T2D in the case-control cohort, nor were any of the SNP alleles over-transmitted in the TDT analysis. Certain microsatellite alleles, however, indicated a trend for excess transmission to diabetic patients and marginal association with triglyceride levels, FFA and fasting glucose, as well as first phase insulin response and insulin deposition index in model analysis [12].

## Methods

### Case-control subjects

All subjects were of French Caucasian ancestry. Individuals identified by Sladek *et al. *[13] to lie outside the HapMap CEU ancestry cluster were excluded from the study. Type 2 diabetic case subjects were known diabetic patients receiving treatment for the condition. Normoglycaemic control subjects were selected to have a fasting blood glucose concentration <6.1 mM [14]. Case subjects were composed of: (i) 372 probands from diabetic families [3], recruited in Lille; and (ii) 1083 patients with a family history of T2D recruited at the Corbeil-Essonne Hospital. Control subjects were composed of: (i) 353 normoglycaemic parents from T2D families; (ii) 543 subjects from the SUVIMAX (Supplementation en Vitamines et Minéraux Antioxidant) prospective population-based cohort study [15]; and (iii) 742 subjects selected from the DESIR (Data from an Epidemiologic Study on the Insulin Resistance Syndrome) cohort, a large prospective study of insulin resistance in French subjects [16]. Informed consent was obtained from all subjects and the study was approved by the local ethics committees.

### Statistical power

The case-control cohort comprised 1,455 type 2 diabetic subjects (age, 60 ± 12 years; BMI, 29.0 ± 6.0 kg/m^2^; male/female, 56:44%) and 1,638 normoglycaemic subjects (age, 54 ± 13 years; BMI, 24.1 ± 3.3 kg/m^2^; male/female, 43:57%). At α = 0.05, this sample size provided 91% power [17] to detect a type 2 diabetes susceptibility variant, assuming an allele frequency of 0.20, a disease prevalence of 0.1 and a heterozygote relative risk of 1.2 (multiplicative model).

### *APOA2 *tag SNP selection

The genomic target region for tag SNP selection extended across the 3 kb NCBI36 *APOA2 *locus (chr1:159,458,001..159,461,000). A total of three HapMap phase II pairwise tagging SNPs (rs6413453, rs5085 and rs5082) were sufficient to tag the common variation across the *APOA2 *locus (HapMap Data Release 22/Apr07) with r^2 ^and minor allele frequency (MAF) thresholds of 0.9 and 0.05, respectively.

### SNP genotyping

Genotyping was performed with the Sequenom MassARRAY iPLEX system [18]. SNP genotype frequencies were tested for accordance with Hardy-Weinberg equilibrium using chi-squared analysis. Quality control: all genotyped SNPs exhibited a call rate >99% and a Hardy-Weinberg *P *> 0.05, with well defined genotype clusters. In total, 19 case and 42 control subjects (1.97% of the total sample size) consistently failed genotyping and were removed from further analysis.

### Statistical analyses

To test for association of *APOA2 *SNPs with type 2 diabetes, chi-squared analysis of allele and genotype counts was performed. Pairwise SNP linkage disequilibrium (LD) values were calculated from the genotype data of the control cohort with Haploview (4.0) [19]. Quantitative anthropomorphic phenotypes, body mass index (BMI), weight and waist-hip ratio (WHR), as well as fasting serum levels of glucose, insulin, triacylglycerol (TG), total and high-density lipoprotein (HDL)-cholesterol, apolipoprotein A-I (APOA1) and apolipoprotein B (APOB), measured in 1,539 normoglycaemic subjects from the control cohort, were log transformed and adjusted for age, sex and BMI, as appropriate. SNPs were tested for association with adjusted quantitative traits using SPSS 15.0 with the ANOVA test under a codominant model. Quantitative trait association p-values are presented uncorrected for multiple testing. Combined analysis of association datasets was carried out by pooling of log-transformed odds ratios with the inverse variance method for the fixed-effects and random-effects models. The DIAGRAM [20] data included a sib-ship component from the DGI GWAS [21]; however, due to uncertainties about controlling for the effects of the relatedness of part of the study sample, we excluded these subjects and obtained data solely for unrelated subjects from the authors (E. Zeggini, personal communication, 23/04/2008). Inter-study heterogeneity was assessed with Cochran's *Q *statistic and the I^2 ^metric [22]. These calculations were performed using R (2.5.1) statistical software [23].

## Results

None of the three genotyped *APOA2 *tag SNPs showed evidence for association (*P *< 0.05) with type 2 diabetes in this French Caucasian cohort (Table 1; genotype counts see Additional file 1: Additional table 1). This finding is in agreement with the French GWAS [13], as well as a recent meta-analysis [20] of the DGI [21], FUSION [24] and WTCCC [25] genome-wide association studies.

**Table 1:** Association of *APOA2 *SNPs with type 2 diabetes.

**SNP**	**Allele**^a^	**Chr Position (NCBI36)**	**Gene Region**	**N subjects**		**Allele 1 (%)**	**Allele 2 (%)**	**Call Rate**	**OR (95% CI)**	***P***	**DIAGRAM GWAS-MA** ***P***^#^
**rs6413453**	C/T	159458940	INTRONIC	T2D	1414	2523 (89)	299 (11)	>99%	1.04 (0.89–1.23)	0.619	0.808^$^
				NG	1581	2809 (89)	347 (11)	>99%			
**rs5085**	G/C	159459135	INTRONIC	T2D	1441	2304 (80)	568 (20)	100%	1.08 (0.95–1.23)	0.245	0.225
				NG	1596	2595 (81)	593 (19)	>99%			
**rs5082**	T/C	159460307	UPSTREAM	T2D	1442	1796 (63)	1066 (37)	>99%	1.03 (0.93–1.14)	0.591	0.408^$^
				NG	1603	1963 (62)	1199 (38)	>99%			

^a^SNP alleles are shown as major/minor. ^#^Please refer to Methods section for details. ^$^Imputed SNPs.

### Quantitative trait associations

The *APOA2 *promoter SNP rs5082 was marginally associated with total cholesterol levels (*P *= 0.026) and waist-to-hip ratio (*P *= 0.029) (Table 2), and both associations exhibited a linear increase of the value of the quantitative phenotype from the common homozygote to the rare homozygote allele carriers (Table 3). The intronic variant rs6413453 was marginally associated with weight (*P *= 0.049), but given the small number of homozygote carriers of the minor allele (n = 13), it is likely that this is a spurious result (Table 2).

**Table 2:** Association of *APOA2 *SNPs with quantitative traits in French Caucasian control subjects.

Trait	n	Mean ± SD	rs6413453	rs5085	rs5082
BMI	1437	24.37 ± 0.09	0.682	0.806	0.075
GLY0	1400	5.21 ± 0.02	0.820	0.401	0.687
INS0	843	50.01 ± 1.17	0.632	0.280	0.450
TG	1311	1.12 ± 0.02	0.178	0.713	0.404
CHOL_TOT	1318	5.94 ± 0.03	0.509	0.249	**0.026**
HDL	792	1.58 ± 0.01	0.658	0.171	0.913
APOA1	1427	1.68 ± 0.01	0.169	0.219	0.778
APOB	1425	1.1 ± 0.01	0.829	0.518	0.121
WEIGHT	1191	66.14 ± 0.33	**0.049**	0.389	0.603
WHR	1164	0.84 ± 0.00	0.932	0.341	**0.029**

Association testing of *APOA2 *SNPs was carried out by ANOVA using SPSS (15.0). All traits were log transformed prior to correction for age, sex and BMI by linear regression, as appropriate. Trait means are presented anti-logged and *P*-values are reported without correction for multiple testing. BMI: body mass index; GLY0: fasting glucose; INS0: fasting insulin; TG: triacylglycerol; CHOL_TOT: total cholesterol; HDL: high-density lipoprotein-cholesterol; APOA1: apolipoprotein A-I; APOB: apolipoprotein B; WHR: waist-hip ratio.

**Table 3:** Distribution of quantitative trait values grouped by genotype for significant association results.

	Genotypes (n)			
	
rs5082	TT	TC	CC	*P*
CHOL_TOT (mM)	5.88 ± 0.97 (506)	5.95 ± 0.92 (600)	6.10 ± 1.01 (185)	0.026
WHR	0.841 ± 0.091 (455)	0.843 ± 0.092 (529)	0.855 ± 0.092 (153)	0.029
rs6413453	CC	CT	TT	
WEIGHT (kg)	66.02 ± 11.60 (905)	66.10 ± 11.52 (229)	70.38 ± 8.63 (13)	0.049

Association testing was carried out by ANOVA comparison of trait means between genotype groups using SPSS (15.0). All traits were transformed prior to correction for age, sex and BMI by linear regression. The quantitative trait values displayed are the anti-logged trait means ± SD.

### Linkage disequilibrium structure of the *APOA2 *genomic locus

The structure of linkage disequilibrium across the three tag SNPs studied was consistent with HapMap phase II data. The levels of intermarker LD (r^2^) were negligible for the *APOA2 *locus (Additional file 1: Additional table 2). Therefore, no haplotype analysis was performed for *APOA2 *variants.

### Meta-analysis of *APOA2 *variants

Meta-analysis combining publicly available data with our current study confirmed in over 12,000 subjects that none of the *APOA2 *tag SNPs were associated with type 2 diabetes (Additional files 2, 3, 4) and there appeared to be no study heterogeneity (Cochran's *Q*: *P *> 0.72; I^2 ^= 0% for all variants). Consistent with the absence of inter-study heterogeneity the fixed and random effects models were identical for all three variants, therefore only the fixed effects are displayed.

## Discussion

The *APOA2 *gene appeared to be a highly plausible candidate gene for type 2 diabetes susceptibility. In addition to defining the peak of linkage at chromosome 1q in French and Utah Caucasian families, *APOA2 *is the second most abundant lipoprotein component of HDL-cholesterol and has been implicated in the regulation of lipid metabolism and insulin sensitivity.

Three HapMap phase II tag SNPs suffice to cover the common variation spanning *APOA2 *and genotyping of these variants demonstrated no association with type 2 diabetes. However, we cannot rule out the possibilities that distal common variants or rare *APOA2 *variants may be associated with type 2 diabetes. The quantitative trait association for the *APOA2 *variant rs5082 with total cholesterol levels was consistent with the physiological role of apoA-II; however, the strength of the association was marginal, as were the remaining, weaker associations with other continuous traits.

Meta-analysis of our data with DIAGRAM [20] consortium data, which was a mixture of directly genotyped and imputed SNPs (Table 1), corroborated our findings of no association with type 2 diabetes. It was also welcome to note that the data from imputed SNPs were in this context indistinguishable from that of the directly genotyped variants.

## Conclusion

Using a case-control cohort of over 3,000 subjects, we tested HapMap phase II tag SNPs that capture the common variation spanning the *APOA2 *genomic locus for association with type 2 diabetes, but failed to find any evidence supporting a contribution of this locus to type 2 diabetes susceptibility. This finding was confirmed in a combined analysis of more than 12,000 subjects.

## Abbreviations

DGI: Diabetes Genetics Initiative; DIAGRAM: Diabetes Genetics Replication And Meta-analysis; FFA: free fatty acids; FUSION: Finland-United States Investigation of NIDDM Genetics; GWAS: Genome-wide association study; GWAS-MA: Genome-wide association study meta-analysis; WTCCC: Wellcome Trust Case-Control Consortium.

## Competing interests

The authors declare that they have no competing interests.

## Authors' contributions

KD participated in the design of the study, carried out the SNP genotyping, performed the analysis of the genotype data and drafted the manuscript. GC, MM, JT, SH and BB contributed to the design of the study. PF contributed to the study design and to drafting of the manuscript. FG contributed to the design of the study, the analysis of the genotype data and in drafting the manuscript. All authors read and approved the final manuscript.

## Pre-publication history

The pre-publication history for this paper can be accessed here:



## Supplementary Material

Additional file 1 **Additional tables 1 and 2.** Word document containing a a table listing the genotype counts for each SNP and a tabular representation of the LD structure across the locus. Click here for file

Additional file 2 **Forest plot graphical summary of the meta-analysis for SNP rs6413453.** Published data from the DIAGRAM [20] GWAS meta-analysis were combined with our data by pooling of log-transformed odds ratios with the inverse variance method using R (2.5.1) [23] software. Click here for file

Additional file 3 **Forest plot graphical summary of the meta-analysis for SNP rs5085.** Published data from the DIAGRAM [20] GWAS meta-analysis were combined with our data by pooling of log-transformed odds ratios with the inverse variance method using R (2.5.1) [23] software. Click here for file

Additional file 4 **Forest plot graphical summary of the meta-analysis for SNP rs5082.** Published data from the DIAGRAM [20] GWAS meta-analysis were combined with our data by pooling of log-transformed odds ratios with the inverse variance method using R (2.5.1) [23] software. Click here for file
