# Supplementary material for: Evaluating the association of common APOA2 variants with type 2 diabetes
Source: BMC Med Genet. 2009 Feb 13;10:13. doi: 10.1186/1471-2350-10-13 (PMC2650681; doi:10.1186/1471-2350-10-13)
Supplement: Additional file 1 — Additional tables 1 and 2. Word document containing a a table listing the genotype counts for each SNP and a tabular representation of the LD structure across the locus. [file 1471-2350-10-13-S1.doc]

## Additional Table 1 – *APOA2* SNP genotype counts

| SNP |  | 1/1 | 1/2 | 2/2 | *P* |
| --- | --- | --- | --- | --- | --- |
| rs6413453 | T2D | 1130 | 267 | 17 | 0.651 |
|  | NG | 1248 | 317 | 16 |  |
| rs5085 | T2D | 930 | 453 | 58 | 0.291 |
|  | NG | 1051 | 497 | 48 |  |
| rs5082 | T2D | 576 | 660 | 206 | 0.702 |
|  | NG | 617 | 755 | 231 |  |

T2D: type 2 diabetic cases. NG: normoglycaemic controls. *P*-values are shown for the chi-squared analysis of the genotype counts.

## Additional Table 2 – Linkage disequilibrium structure of the *APOA2* locus.

| **D’**  **r^2^** | **rs6413453** | **rs5085** | **rs5082** |
| --- | --- | --- | --- |
| **rs6413453** |  | 1 | 1 |
| **rs5085** | 0.028 |  | 0.992 |
| **rs5082** | 0.075 | 0.138 |  |

Pairwise linkage disequilibrium between the genotyped markers in the French controls subjects was calculated with Haploview (4.0) software [19].
