# Supplementary figures and images for: Evaluating the association of common APOA2 variants with type 2 diabetes
Source: BMC Med Genet. 2009 Feb 13;10:13. doi: 10.1186/1471-2350-10-13 (PMC2650681; doi:10.1186/1471-2350-10-13)

rs6413453

Current

DIAGRAM

Fixed effects P = 0.649

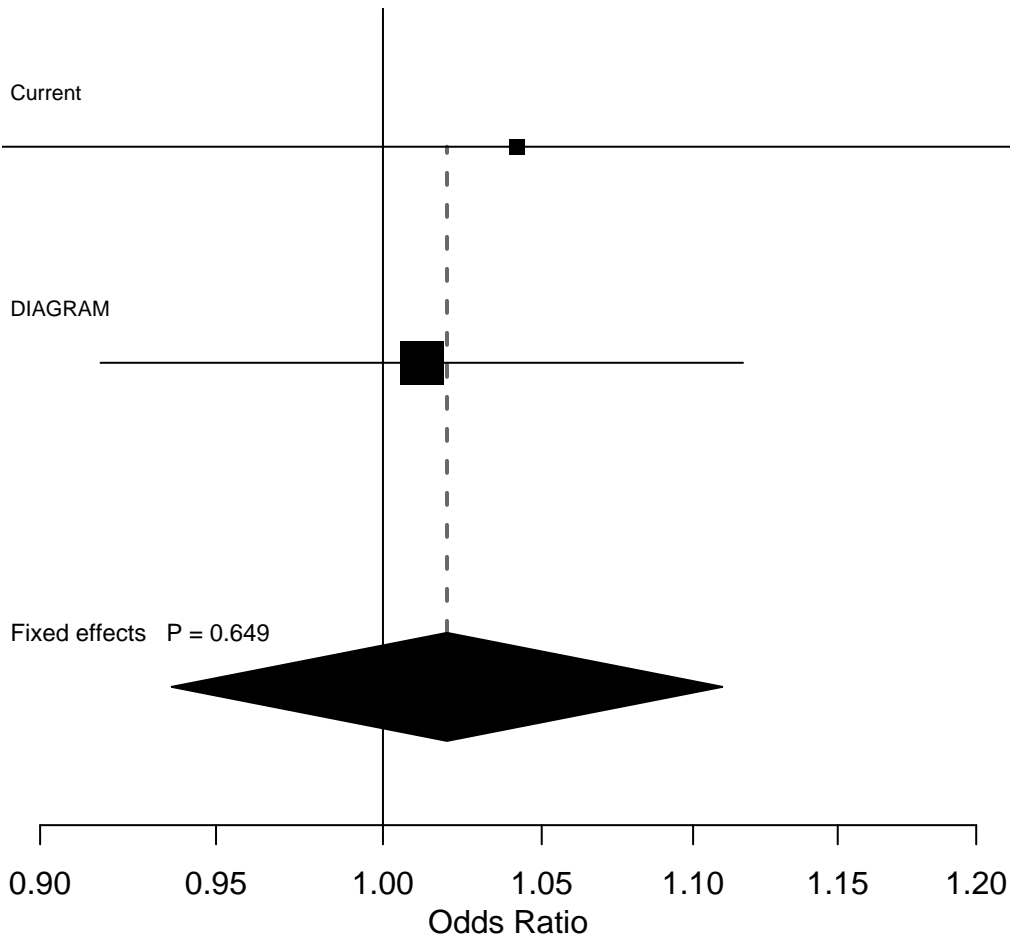

Supplement: Additional file 2 — Forest plot graphical summary of the meta-analysis for SNP rs6413453. Published data from the DIAGRAM [20] GWAS meta-analysis were combined with our data by pooling of log-transformed odds ratios with the inverse variance method using R (2.5.1) [23] software. [file 1471-2350-10-13-S2.pdf]

rs5085

Current

DIAGRAM

Fixed effects P = 0.102

0.90 0.95 1.00 1.05 1.10 1.15 1.20

Odds Ratio

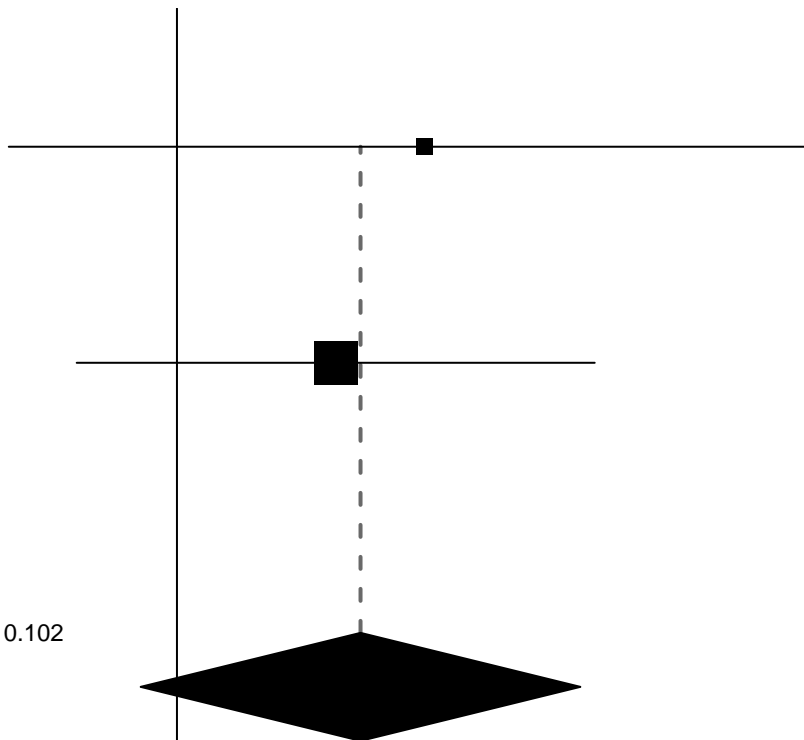

Supplement: Additional file 3 — Forest plot graphical summary of the meta-analysis for SNP rs5085. Published data from the DIAGRAM [20] GWAS meta-analysis were combined with our data by pooling of log-transformed odds ratios with the inverse variance method using R (2.5.1) [23] software. [file 1471-2350-10-13-S3.pdf]

rs5082

Current

DIAGRAM

Fixed effects P = 0.333

0.90

0.95

1.00

1.05

1.10

1.15

1.20

Odds Ratio

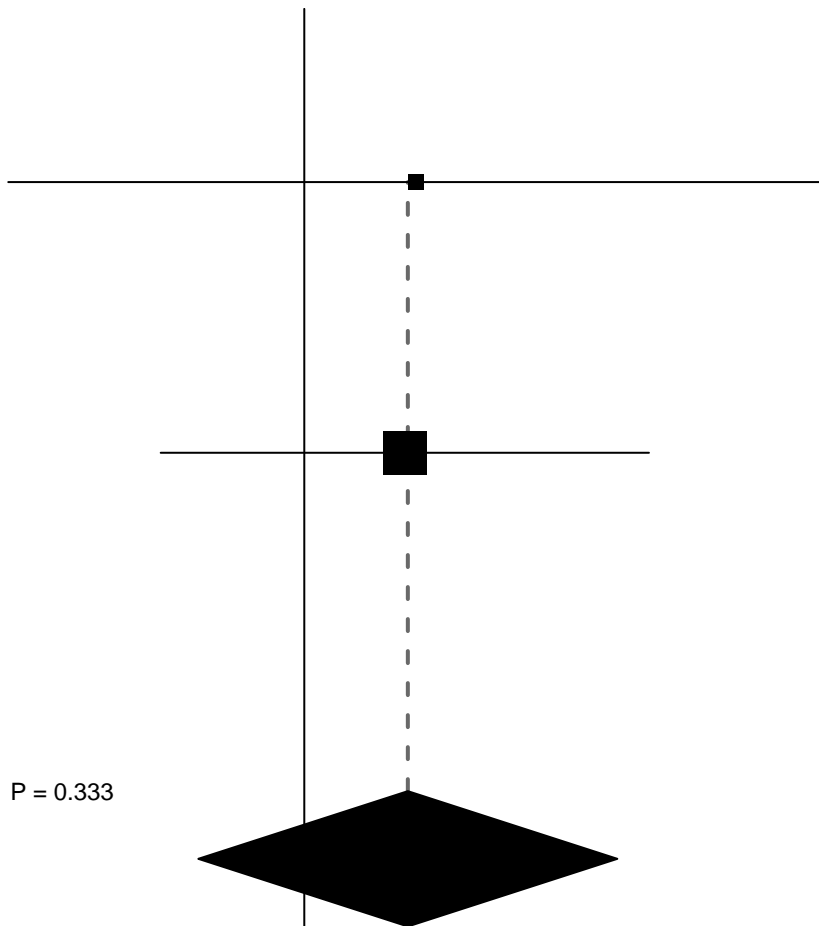

Supplement: Additional file 4 — Forest plot graphical summary of the meta-analysis for SNP rs5082. Published data from the DIAGRAM [20] GWAS meta-analysis were combined with our data by pooling of log-transformed odds ratios with the inverse variance method using R (2.5.1) [23] software. [file 1471-2350-10-13-S4.pdf]
